# Supplementary material for: Innate scavenger receptor-A regulates adaptive T helper cell responses to pathogen infection
Source: Nat Commun. 2017 Jul 11;8:16035. doi: 10.1038/ncomms16035 (PMC5508227; doi:10.1038/ncomms16035)
Supplement: Supplementary Information [file ncomms16035-s1.pdf]

Title of file for HTML: Supplementary Information
Description: Supplementary Figures and Supplementary Table

Title of file for HTML: Peer Review File
Description:

Innate Scavenger Receptor-A regulates adaptive T helper cell responses to pathogen infection

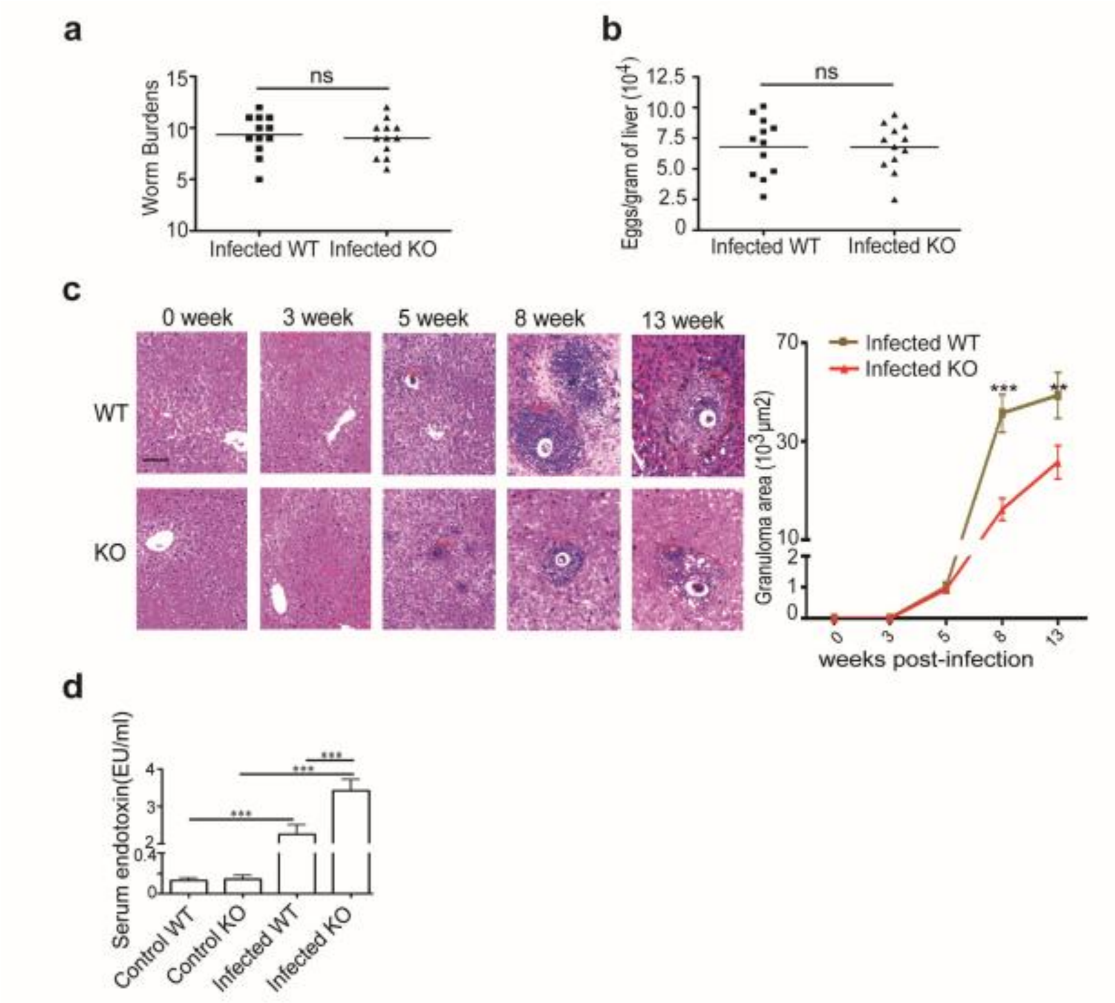

Supplementary Figure 1, Related to Figure 1: SR-A deficiency attenuates immunopathology after *S. japonicum* infection

(**a**) Eight weeks after challenge infection, mice were sacrificed and perfused to collect adult worms. (**b**) The numbers of eggs extracted from the livers were determined by microscopic examination. Data are expressed as the mean ± SD of 12 mice for each group, and the experiments were repeated twice with similar results. ns, not significant (Student's t-test), compared to infected WT mice. (**c**) Mice were randomly chosen and sacrificed at 0 (before infection), 3 (before produce eggs), 5, 8 or 13 weeks post-infection (n=12 each time point per group). Paraffin-embedded liver sections were stained with H&E. Scale bars, 100μm. (**d**) Levels of serum endotoxin in WT and SR-A-deficient mice 8 weeks post infection were determined. Values are given as mean ± SD for each group, and are representative of one typical experiment out of three. $^{**}p < 0.01$, $^{***}p < 0.001$ (ANOVA/LSD).

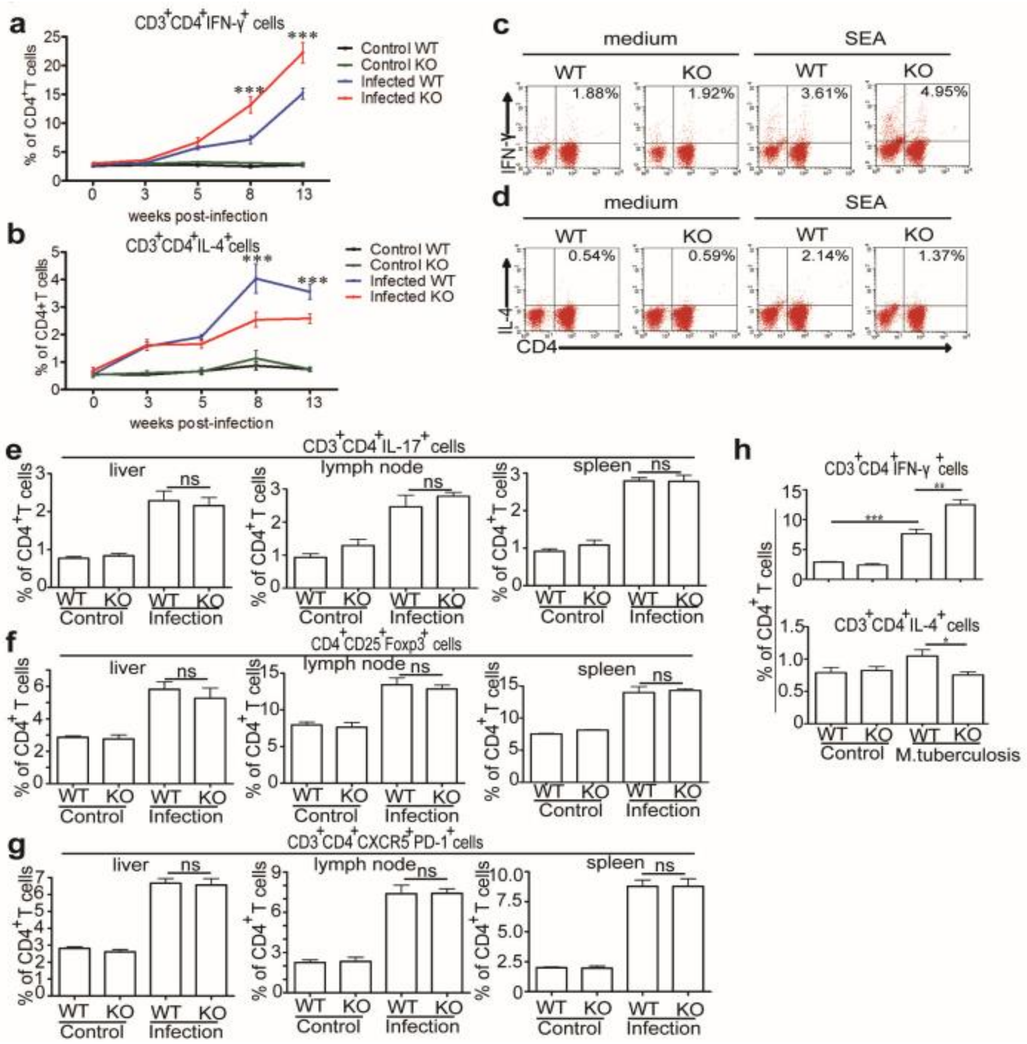

Supplementary Figure 2, Related to Figure 2: SR-A deficiency promotes Th1 responses in *S. japonicum*-infected mouse model

At different time points (0, 3, 5, 8, 13 weeks) after infection, single cell suspensions of mouse livers, mesenteric LN, and spleens from WT or SR-A-deficient mice infected with or without *S. japonicum* were prepared. Cells were stained with CD3-APC and CD4-FITC, and then intracellularly stained with PE-conjugated antibodies against IFN-γ or IL-4 for FACS analysis of $CD3^{+}CD4^{+}IFN\text{-}\gamma^{+}$ (**a**) or $CD3^{+}CD4^{+}IL\text{-}4^{+}$ (**b**) cells, respectively. Data shown were gated on $CD3^{+}CD4^{+}$ cells. Data are expressed as the mean ± SD of 12 mice for each group, all experiments were repeated three times with similar results. Splenocytes from WT or SR-A-deficient mice stimulated with or without SEA, and the proportion of $CD3^{+}CD4^{+}IFN\text{-}\gamma^{+}$ (**c**) or $CD3^{+}CD4^{+}IL\text{-}4^{+}$ (**d**) cells in $CD4^{+}$ T cells was analyzed by FACS. Livers, mesenteric LN, and spleens from WT and SR-A-deficient mice infected with or without *S. japonicum* were harvested. Cells were analysis of Th17 (**e**), Treg (**f**), or Tfh (**g**) cells, respectively. Data are expressed as the mean ± SD of 12 mice for each group, all experiments were repeated three times with similar results, ***p < 0.001, **p < 0.01, * p < 0.05 (ANOVA/LSD). WT and SR-A-deficient mice were injected with heat-inactivated *M. tuberculosis* (i.p. 4mg/kg) (**h**). After 24h, Th1 and Th2 cells in splenocytes were analyzed, respectively. Data are expressed as the mean ± SD of 10 mice for each group, and the experiments were repeated twice with similar results. **p < 0.01, * p < 0.05 (ANOVA/LSD).

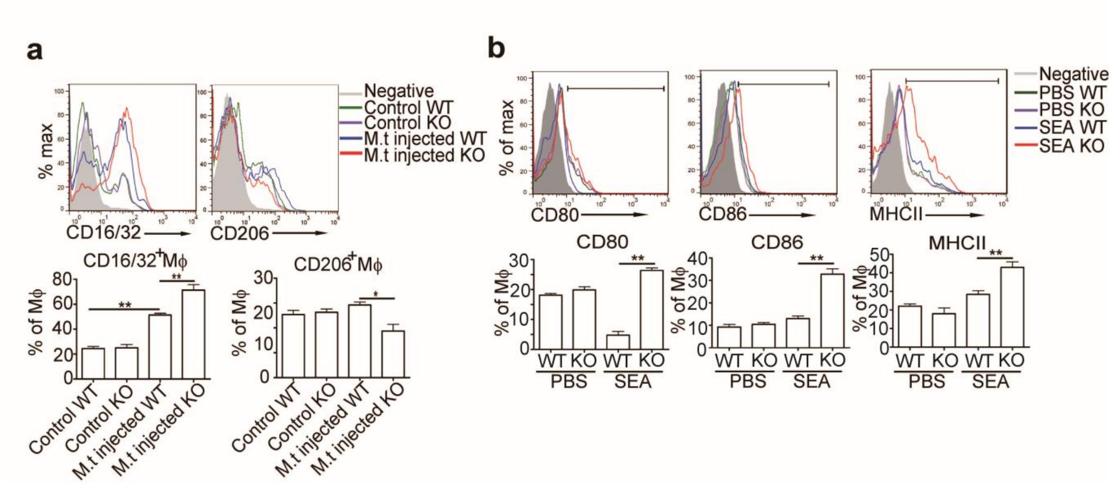

Supplementary Figure 3, Related to Figure 3: SR-A deficiency enhances M1 polarization in heat-inactivated *M. tuberculosis*-immunized mouse models

(**a**) Peritoneal macrophages were isolated from WT and SR-A-deficient mice immunized with heat-inactivated *M. tuberculosis* and analyzed of CD16/32 and CD206 expression, respectively. (**b**) Peritoneal macrophages from control WT or SR-A-deficient mice were stimulated with SEA and analyzed the levels of MHC II, CD80, and CD86 on macrophages by FACS. Data are expressed as the mean ± SD of 12 mice for each group. All experiments were repeated twice with similar results. ** p <0.001, *p<0.05 (ANOVA/LSD).

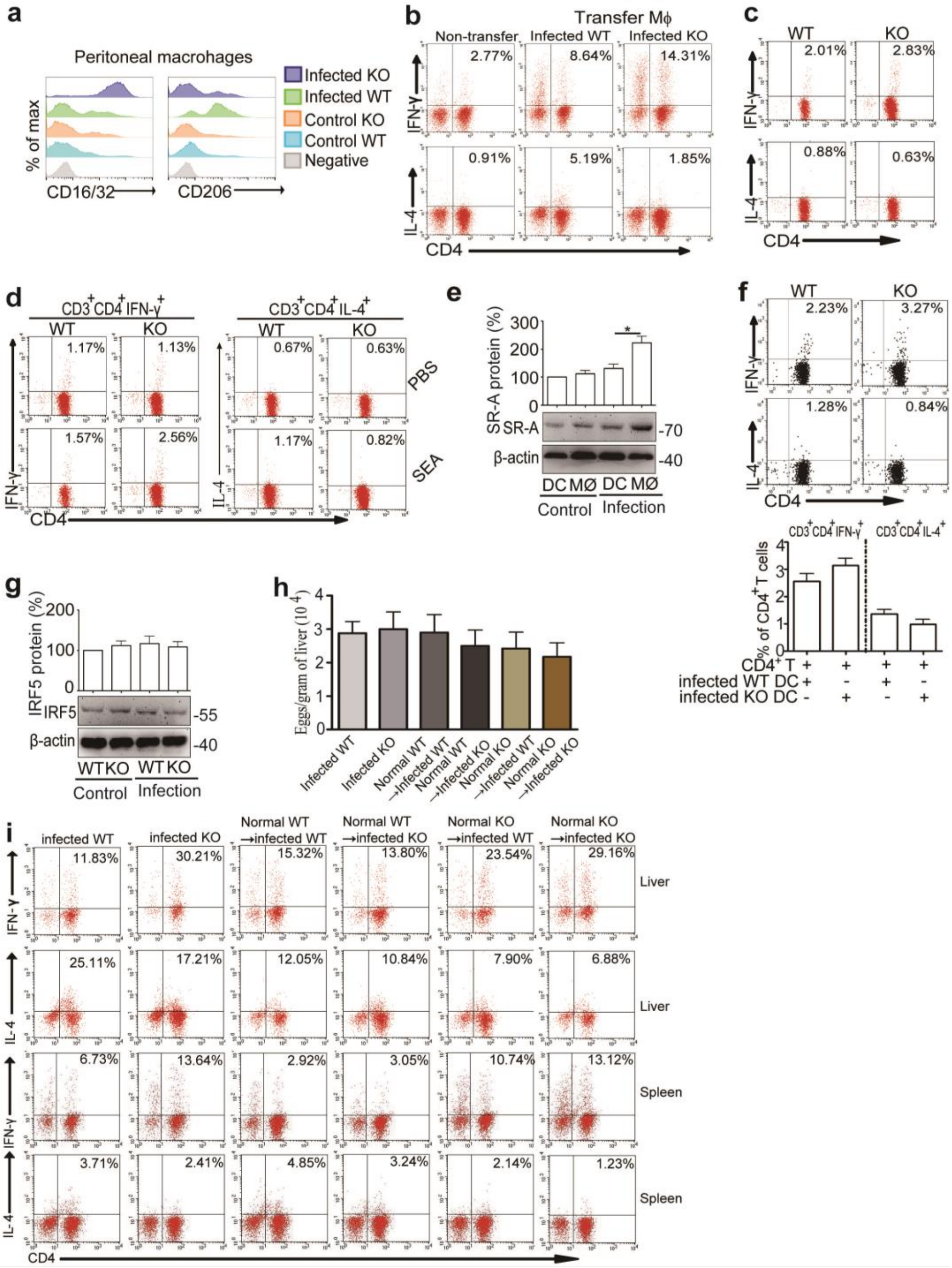

Supplementary Figure 4, Related to Figure 4: SR-A suppressed Th1 but induced Th2 differentiation through regulation of macrophage but not DC responses

(**a**) FACS analysis of proportion of CD16/32^{+} or CD206^{+} on peritoneal macrophages from WT or SR-A deficient mice infected with or without *S. japonicum*. (**b**) Two weeks after adoptive transfer of infected WT or SR-A-deficient mice derived peritoneal macrophages to normal WT mice, representative of Th1 or Th2 cells in recipient mouse splenocytes were shown. (**c**) Representative Th1 or Th2 cells after co-culture of infected mice derived SR-A WT or KO macrophages with normal mice derived CD4^{+} T cells for 48h were shown. (**d**) CD4^{+} T cells from control WT mice were co-cultured with purified peritoneal macrophages from control WT or control SR-A-deficient mice in the presence of SEA, and representative Th1 or Th2 cells were shown. Data are representative of three independent

experiments. DCs of mouse spleens from WT and SR-A-deficient mice infected with or without *S. japonicum* were purified for immunoblot analysis of SR-A (**e**) or IRF5 (**g**) protein. (**f**) $CD4^+$ T cells from control WT mice were purified and co-cultured with purified $CD11c^+$ DCs from infected WT or SR-A-deficient mice for analysis of Th1/Th2 cells. Data are representative of three independent experiments. * $p<0.05$ (ANOVA/LSD). (**h-i**) 4 weeks after adoptive transfer of normal WT or SR-A-deficient mice derived peritoneal macrophages to infected WT or infected SR-A-deficient mice, respectively. (**h**)The numbers of eggs extracted from the livers were determined by microscopic examination. (**i**) Representative Th1 or Th2 cells in the liver or spleen of recipient mouse were shown. Data are expressed as the mean ± SD of 6 mice for each group, and the experiments were repeated twice with similar results. * $p<0.05$, ** $p<0.01$, *** $p<0.001$ (ANOVA/LSD).

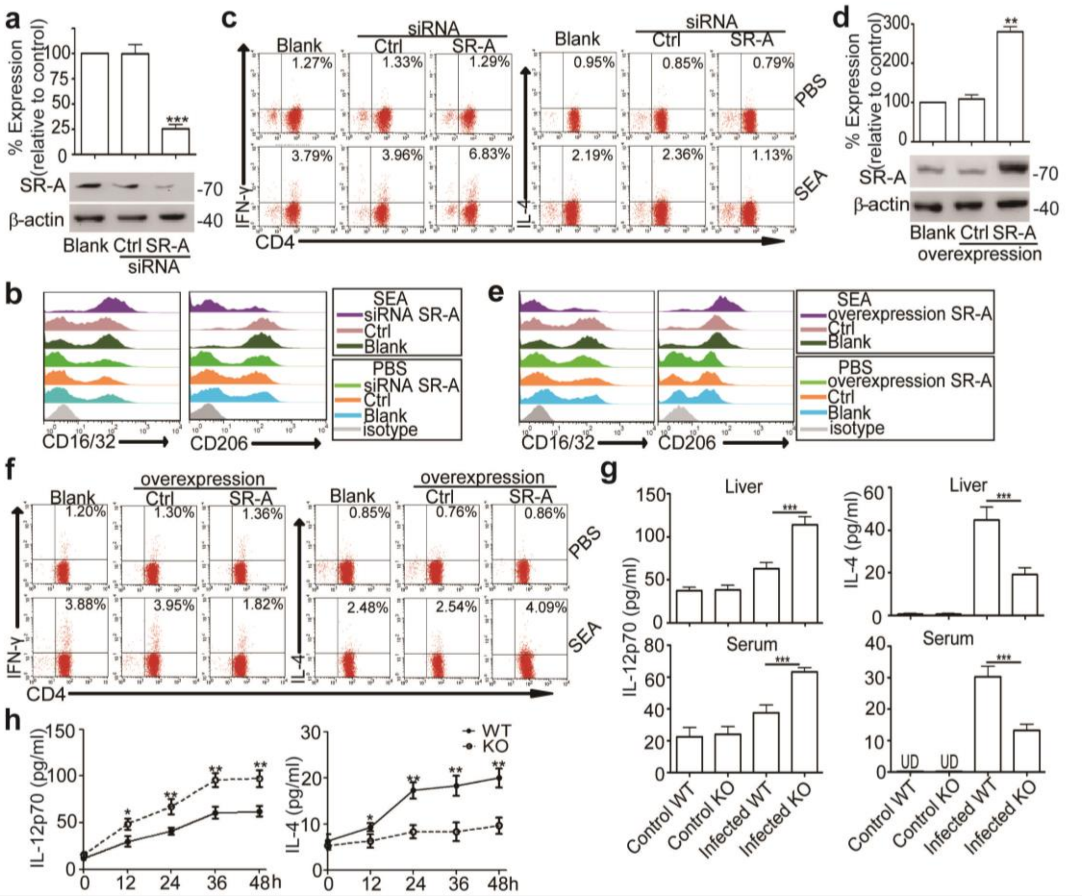

Supplementary Figure 5, Related to Figure 4: SR-A suppressed Th1 but induced Th2 differentiation through regulation of macrophage but not DC responses

(**a**) siRNA targeting SR-A (si-SR-A)-mediated interference efficiently downregulated SR-A expression in BMDMs. Data are expressed as the mean ± SD from three independent experiments, ***p < 0.001. (**b**) BMDMs from control WT mice were transfected with si-SR-A or nontargeting siRNA (ctrl-siRNA) and stimulated with or without SEA, the representative plots of CD16/32^{+} or CD206^{+} cells in BMDMs were analyzed by FACS. And then BMDMs were co-cultured with purified CD4^{+} T cells from control WT mice for Th1/Th2 analysis (**c**). (**d**) pcDNA3.1-MSR-mediated overexpression upregulates SR-A expression in BMDMs. (**e**) BMDMs from control WT mice were transfected with pcDNA3.1-MSR (SR-A) or empty vector (pcDNA3.1) and stimulated with or without SEA, the representative of CD16/32^{+} or CD206^{+} cells in BMDMs were analyzed by FACs. And then BMDMs were co-cultured with CD4^{+} T cells from control WT mice for Th1/Th2 analysis (**f**), data are representative of three independent experiments. **p<0.01 (ANOVA/LSD). (**g**) The protein levels of IL-12p70 or IL-4 in the serum or liver tissue homogenate from WT or SR-A-deficient mice infected with or without *S. japonicum*. Data are expressed as the mean ± SD of 12 mice for each group, all experiments were repeated three times with similar results, UD, undetectable, ***p < 0.001 (ANOVA/LSD). (**h**) ELISA analysis of IL-12p70 or IL-4 produced by peritoneal macrophages from WT or SR-A-deficient mice in the presence or absence of SEA stimulation for 12, 24, 36, and 48h. Data are representative of three independent experiments. * p<0.05 (ANOVA/LSD).

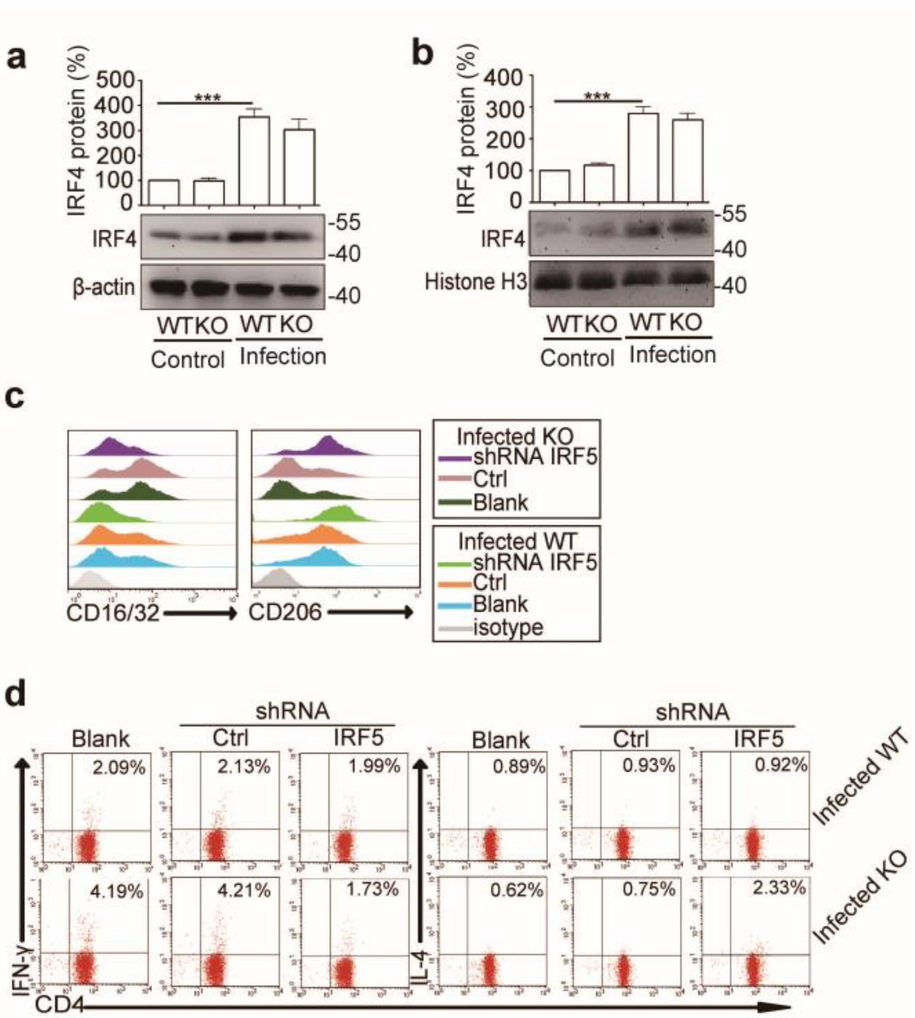

Supplementary Figure 6, Related to Figure 5: SR-A regulates macrophage polarization by regulation of IRF5 but not IRF4

Peritoneal macrophages from *S. japonicum*-infected WT or SR-A-deficient mice were prepared for analysis of IRF4 total (**a**) or nuclear (**b**) protein by immunoblot. Peritoneal macrophages from *S. japonicum*-infected WT or SR-A-deficient mice were transfected with sh-IRF5 or ctrl-shRNA in the presence of SEA. (**c**) Representative plots of CD16/32-expressed macrophages or CD206-expressed macrophages. (**d**) After transfection, peritoneal macrophages were co-culture with $CD4^+$ T cells from control WT mice stimulated with SEA, and representative of Th1 and Th2 cells were shown.

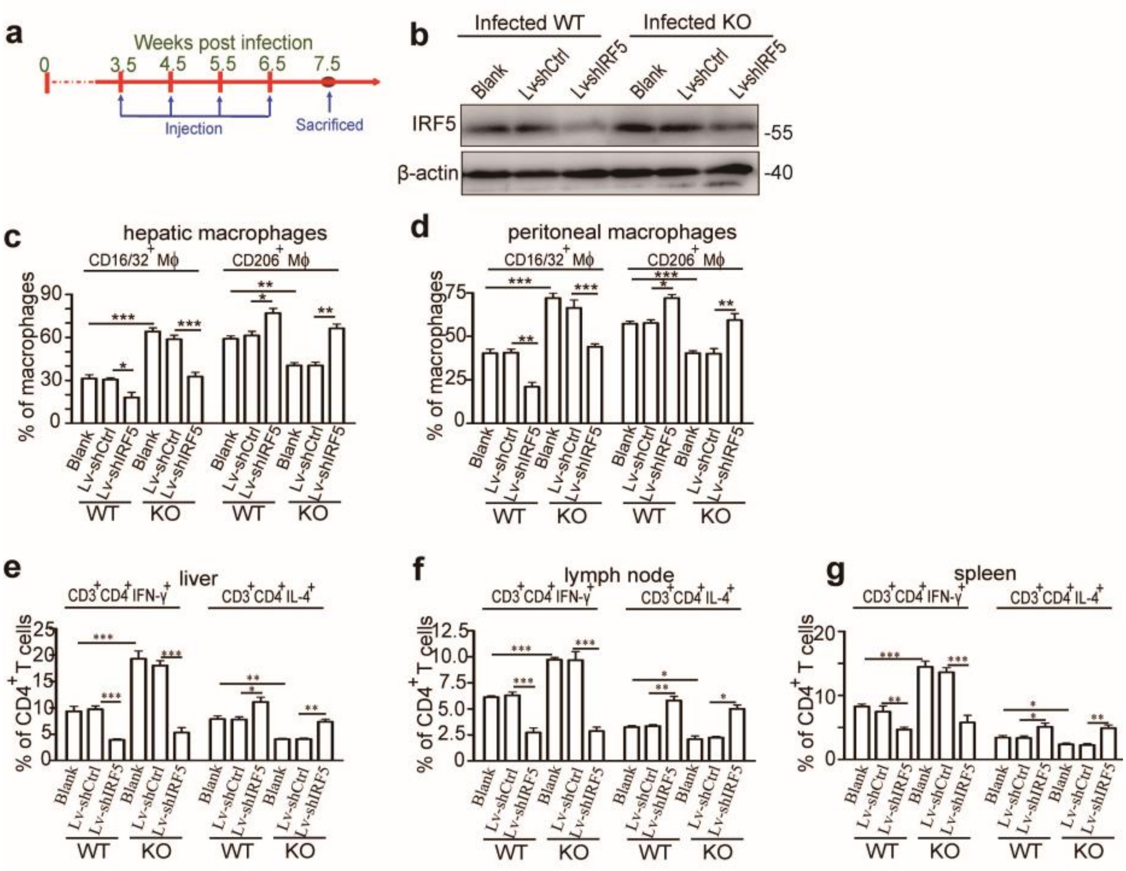

Supplementary Figure 7, Related to Figure 6: Knockdown of IRF5 reduces M1 and Th1 polarization and restores liver pathology in SR-A-deficient mice

(**a**) Mice were intravenously injected with shRNA lentiviral particles targeting IRF5 since 3.5 weeks post *S. japonicum* infection by weekly for 4 weeks and sacrificed at 7.5 weeks post *S. japonicum* infection for further study. (**b**) The efficiency of IRF5 knockdown in liver of *S. japonicum*-infected WT or SR-A KO mice after Lv-shRNA injection on 7.5 wk post-infection. Statistical analysis of $CD16/32^+$ or $CD206^+$ cells in hepatic (**c**) or peritoneal (**d**) macrophages from *S. japonicum*-infected WT or SR-A-deficient mice. Statistical analysis of Th1 or Th2 cells in $CD4^+$ T cells in mouse livers (**e**), mesenteric LN (**f**), and spleens (**g**) from *S. japonicum*-infected WT and SR-A-deficient mice. Data are representative of two experiments with 6 mice per group in each experiment. ***$p < 0.001$, **$p < 0.01$, *$p < 0.05$ (ANOVA/LSD).

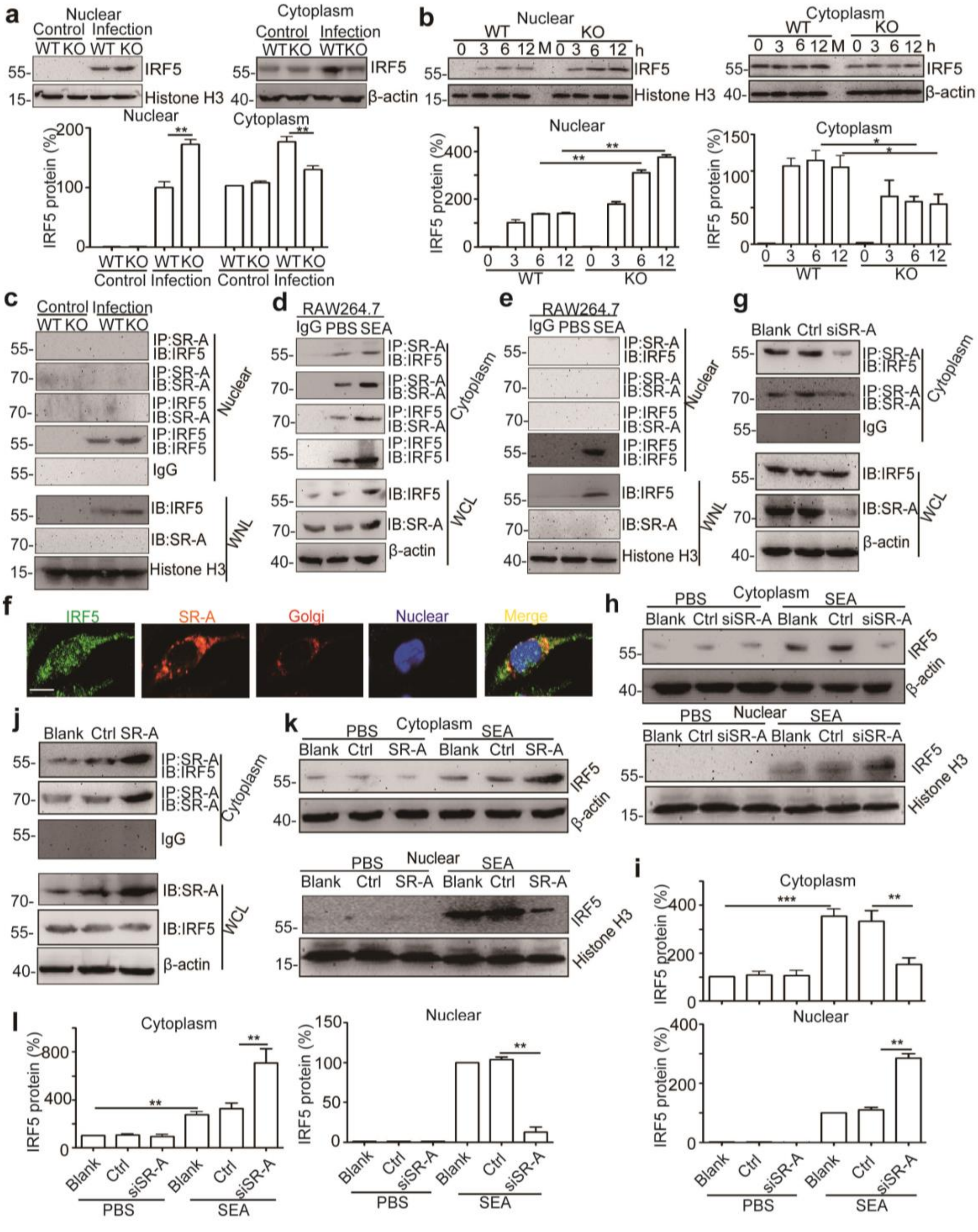

Supplementary Figure 8, Related to Figure 6: SR-A interacts with IRF5 in cytoplasm and inhibits IRF5 nuclear transfer

(**a**) Immunoblot analysis of IRF5 protein in nuclear and cytoplasmic fractions of peritoneal macrophages from WT or SR-A-deficient mice infected with or without *S. japonicum*. (**b**) Immunoblot analysis of IRF5 protein in nuclear and cytoplasmic fractions of peritoneal macrophages for 3, 6, 12h from normal WT or SR-A-deficient mice in the presence of SEA. Immunoblot analysis of IRF5 of endogenous SR-A immunoprecipitated from nuclear (**c**) protein extracted from peritoneal macrophages of WT or SR-A-deficient mice infected with or without *S. japonicum*. The association of SR-A and IRF5 was confirmed by a reciprocal immunoprecipitation

assay using anti-IRF5. Immunoblot was also carried out using whole nuclear lysate (WNL). Immunoblot analysis of IRF5 of endogenous SR-A immunoprecipitated from cytoplasmic (**d**) and nuclear (**e**) protein extracted from RAW264.7 cells stimulation with SEA. Co-localization of endogenous SR-A and IRF5 with Golgi in peritoneal macrophages from control WT mice by confocal microscopy, scale bars, 10μm (**f**). After transfection with si-SR-A (**g-i**) or pcDNA3.1-MSR (**j-l**), immunoblot analysis of IRF5 protein in cytoplasmic and nuclear of RAW264.7 cell fractions with or without SEA. Data are representative of three independent experiments. ***$p < 0.001$, **$p < 0.01$, *$p < 0.05$ (ANOVA/LSD).

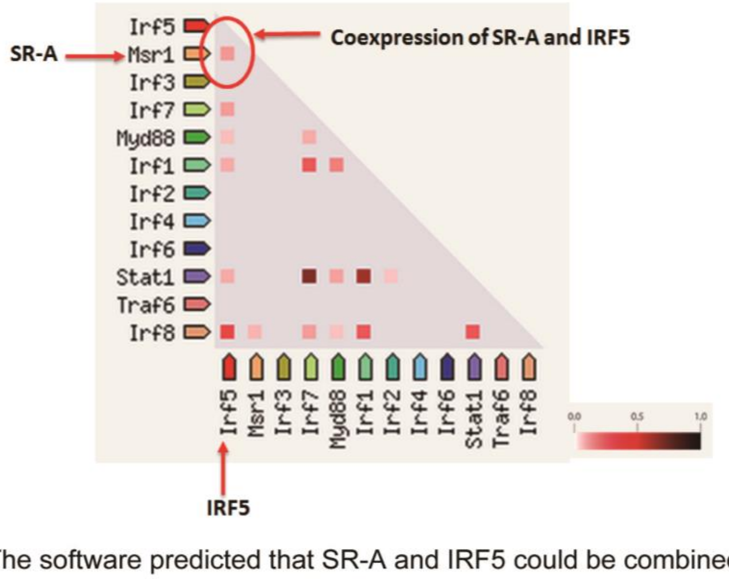

Supplementary Figure 9, Related to Figure 6: prediction of the interaction between SR-A and IRF5

STRING database (http://string.embl.de/) was used to predict the possible interaction of SR-A and IRF5.

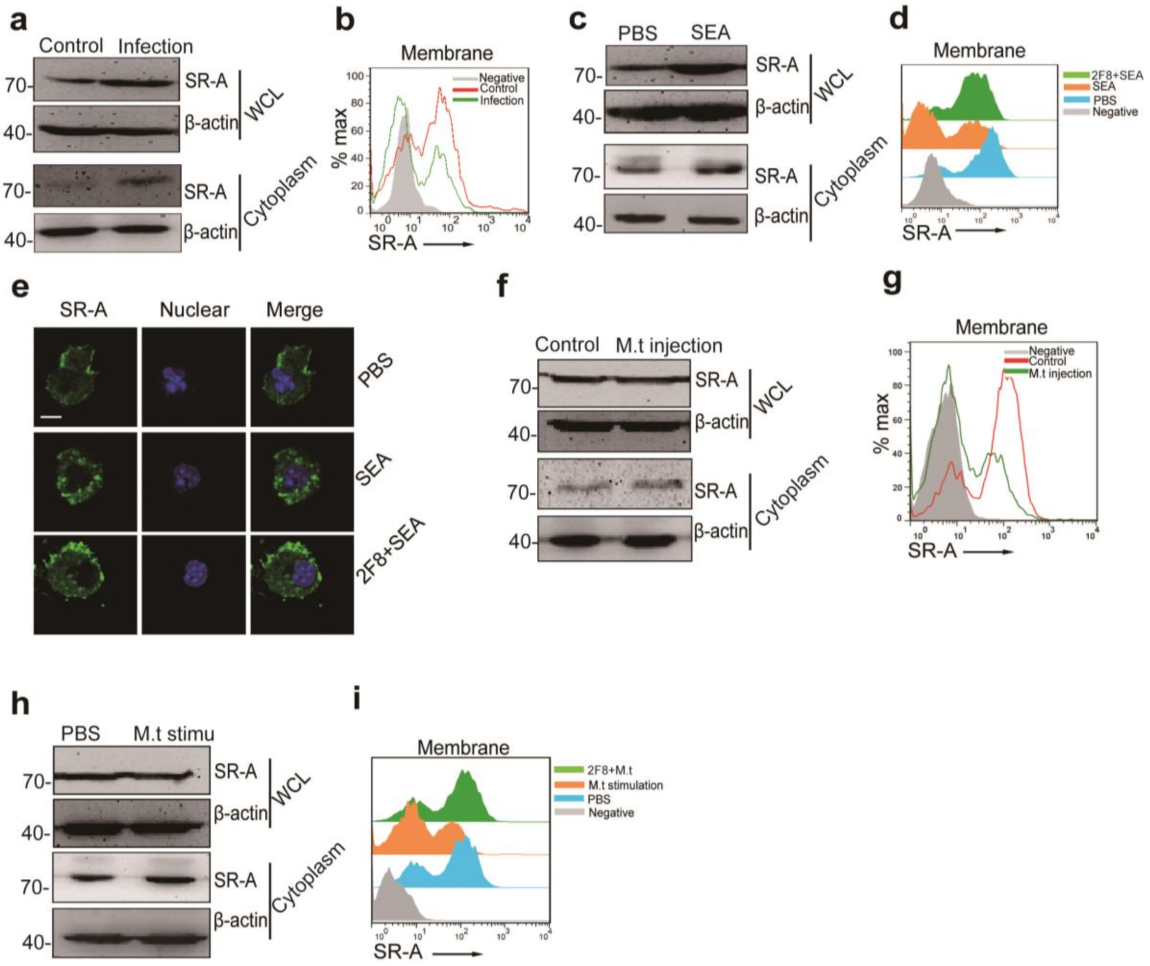

Supplementary Figure 10, Related to Figure 6: SR-A expression is regulated by pathogen infection or stimuli

Peritoneal macrophages were isolated from normal and *S. japonicum*-infected or heat-inactivated *M. tuberculosis* injected WT mice, western blot was carried out for evaluation of SR-A in whole cell lysate (WCL) or cytoplasm (**a,f**), and the levels of membrane SR-A were analyzed by FACS (**b,g**), respectively. (**c,h**) Peritoneal macrophages were *in vitro* stimulated with SEA or heat-inactivated *M. tuberculosis* and total and cytoplasmic SR-A was analyzed by western blot. (**d,e,i**) Peritoneal macrophages were *in vitro* stimulated with PBS, SEA, or heat-inactivated *M. tuberculosis* after pretreatment with SR-A blocking antibody (2F8), then membrane SR-A was analyzed by FACS or confocal microscopy. (Scale bars, 10μm).

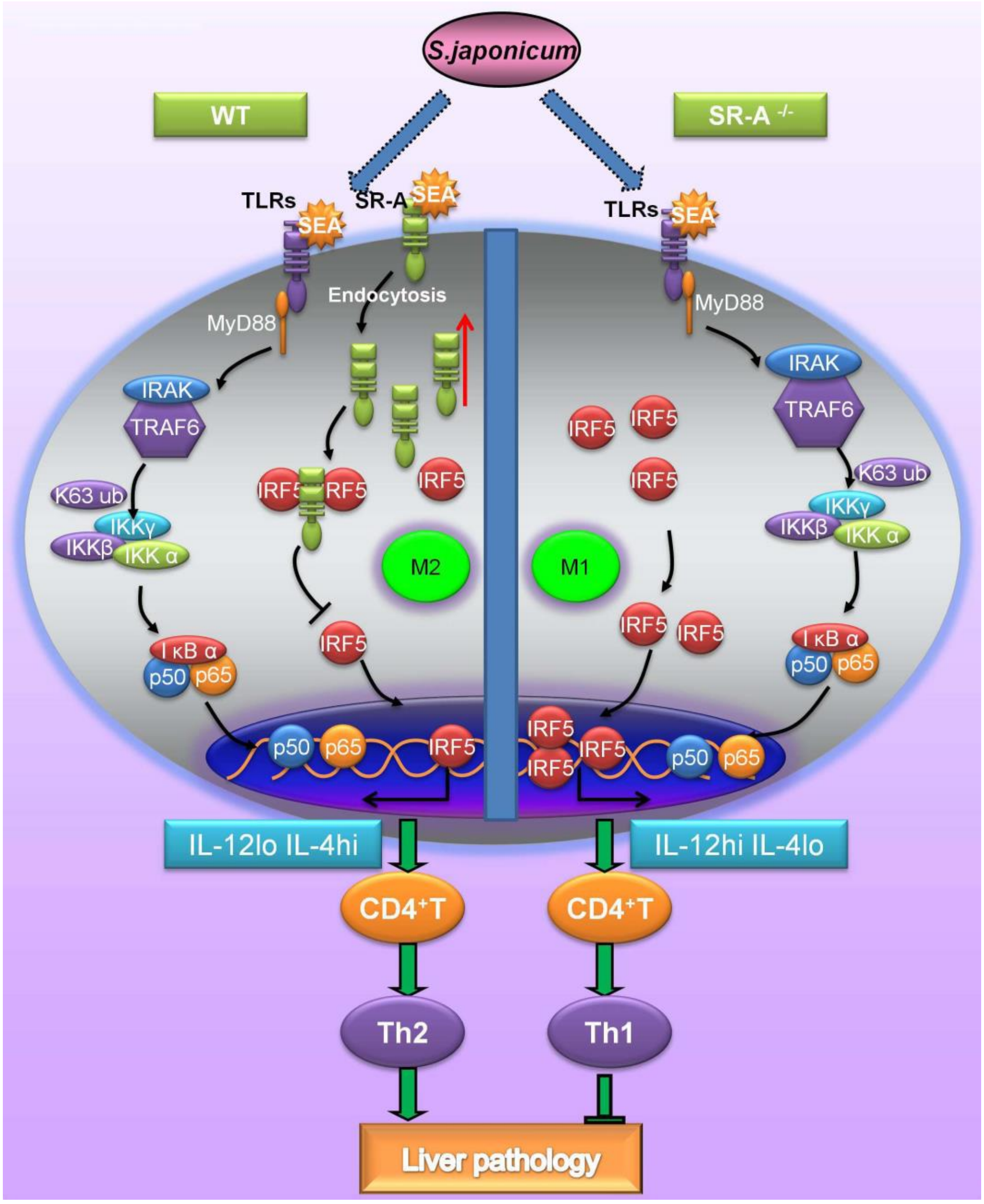

Supplementary Figure 11: Diagram of SR-A regulates of adaptive T helper cell responses

The current study suggests the possible roles of SR-A in regulating IRF5 nuclear translocation. After the pathogen infection or stimuli, SR-A localizes to the plasma membrane and recognizes the antigen (*e.g.*SEA). The receptor is endocytosed with its recognized antigen, and binds to the cytoplasmic IRF5, a master regulator of the macrophage polarization and Th responses. The interaction of SR-A and IRF5 in cytoplasm inhibits IRF5 translocation and activation. This process is characterized by M2 polarization and high IL-4 production, which induced Th2 differentiation and promoting liver pathology. However, SR-A-deficiency resulted in increasing IRF5 nuclear translocation, the increase of nuclear IRF5 shifted macrophage polarization from M2 towards M1, which subsequently switched Th responses from Th2 to Th1.

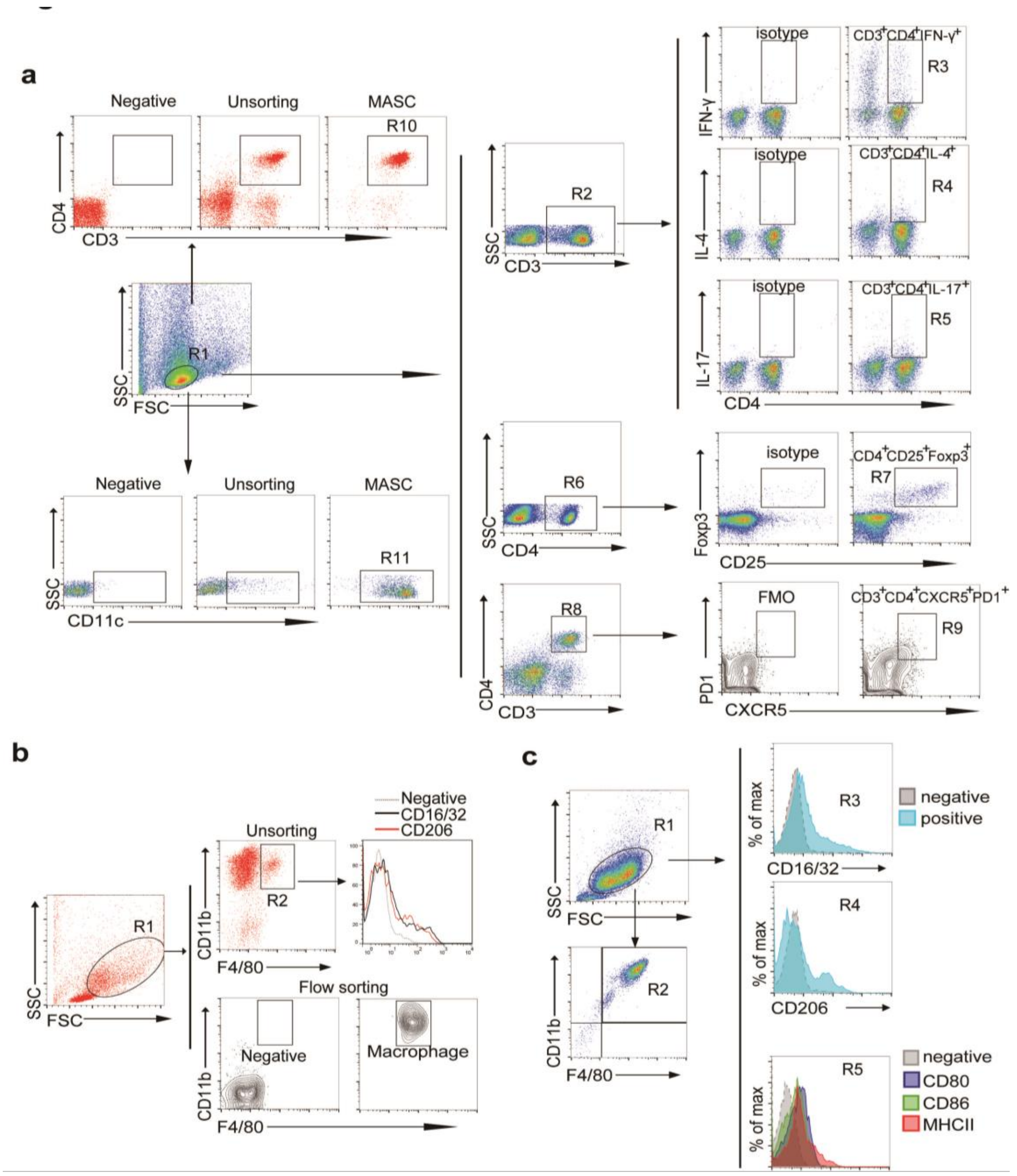

Supplementary Figure 12: FACS gating/sorting strategies

(**a**) Gating schemes for analysis of the percentages of CD3⁺CD4⁺IFN-γ⁺(R3, gated from R1 and R2, related to Figures 2a, 2c, 4a-4c, 4g-4i, 5e, Supplementary Figures 2a, 2c, 2h, 4b-4d, 4f, 4i, 5c, 5f, 6d, 7e-7g), CD3⁺CD4⁺IL-4⁺(R4, gated from R1 and R2, related to Figure 2b, 2d, 4a-4c, 4f-4i, 5e, Supplementary Figures 2b,2d, 2h, 4b-4d, 4f, 4i, 5c, 5f, 6d, 7e-7g), CD3⁺CD4⁺IL-17⁺(R5, gated from R1 and R2, related to Supplementary Figure 2e), CD4⁺CD25⁺Foxp3⁺ (R7, gated from R1 and R6, related to Supplementary Figure 2f), CD3⁺CD4⁺CXCR5⁺PD-1⁺ (R9, gated from R1 and R8, related to Supplementary Figure 2g). Gating schemes for analysis of the percentages of CD3⁺CD4⁺(R10, gated from R1) after purification by magnetic-activated cell sorting (MACS), related to Figures 4b, 4c, 4h-4i, 5e, Supplementary Figures 4c-4d, 4f, 5c, 5f, 6d). Gating schemes for analysis of the percentages of CD11c⁺(R11, gated from R1) after purification by MACS, related to Supplementary Figures 4e-4g). (**b**) Gating schemes for analysis of the percentages of F4/80⁺CD11b⁺CD16/32⁺ (gated from R1 and R2) or

$F4/80^+CD11b^+CD206^+$ (gated from R1 and R2) cells in the liver, related to Figure 3a. Mononuclear cells from livers of WT and SR-A-deficient mice were collected by Percoll gradient centrifugation, and then purified by flow sorting, related to Figure 3c. (**c**) Gating schemes for analysis of the percentages of $CD16/32^+$ (R3, gated from R1) or $CD206^+$ (R4, gated from R1) cells in the peritoneal macrophges or BMDMs, related to Supplementary Figures 3a, 4a, 5b, 5e, and 6c. Gating schemes for analysis of the percentages of $CD80^+$, $CD86^+$, or $MHCII^+$ (R5, gated from R1) cells in the peritoneal macrophges, related to Supplementary Figure 3b.

Supplementary Figure 13: Uncropped scans of western blots displayed in the main figures

Fig5a

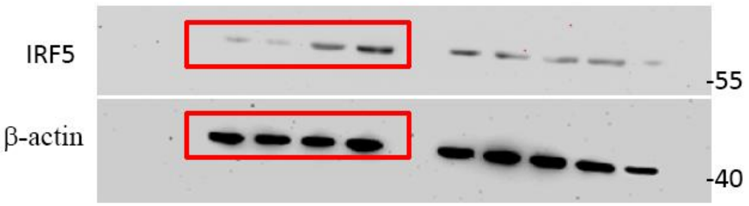

Fig5b

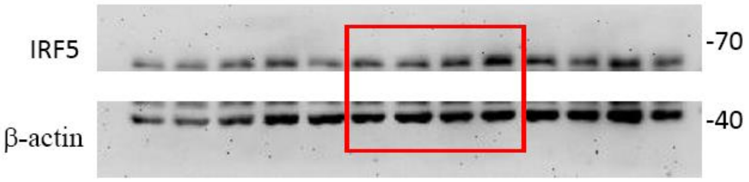

Uncropped scans of western blots displayed in the main figures

Fig6g

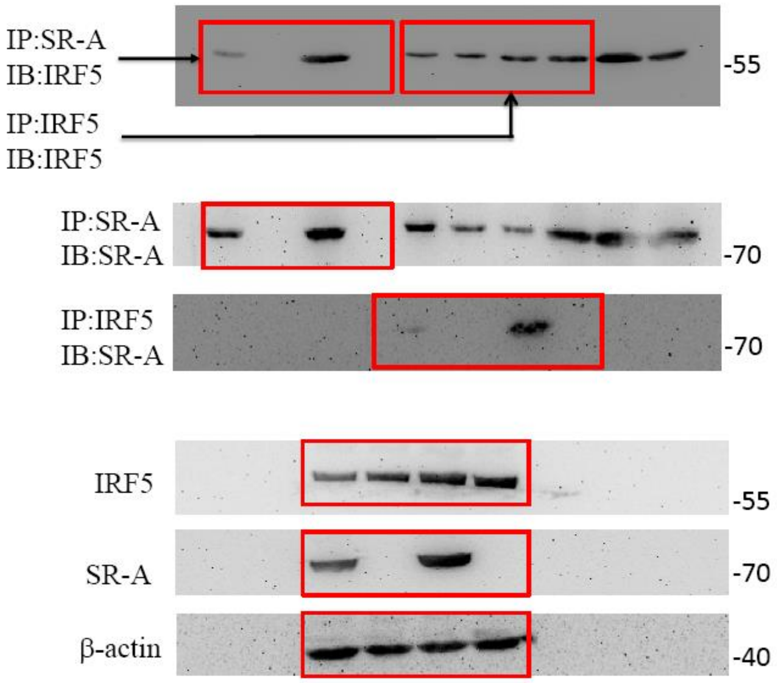

Uncropped scans of western blots displayed in the main figures

FigS4e

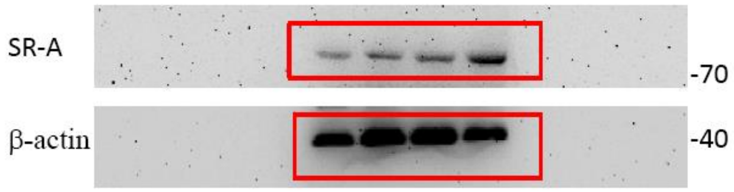

FigS4g

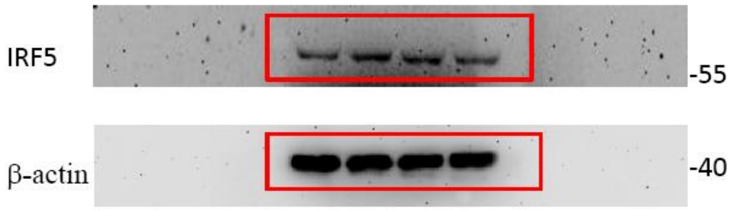

Uncropped scans of western blots displayed in the main figures

FigS5a and FigS5d

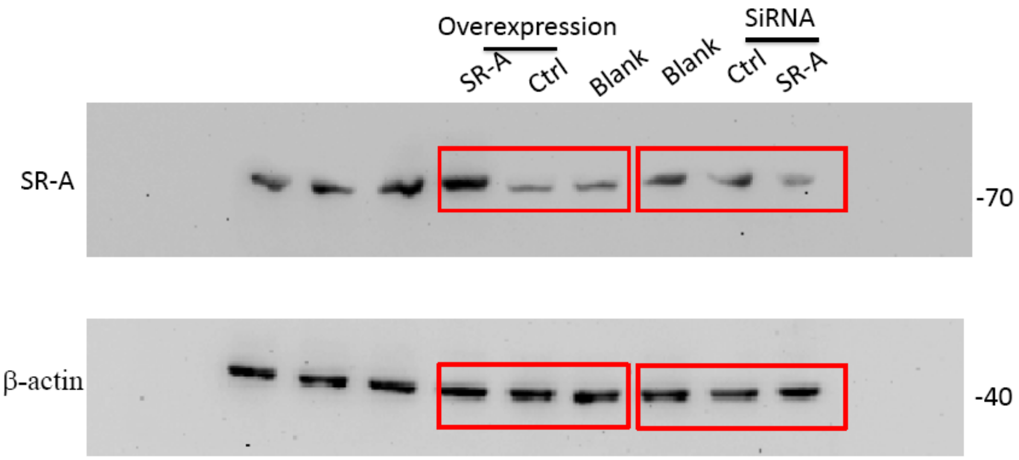

Uncropped scans of western blots displayed in the main figures

FigS6a

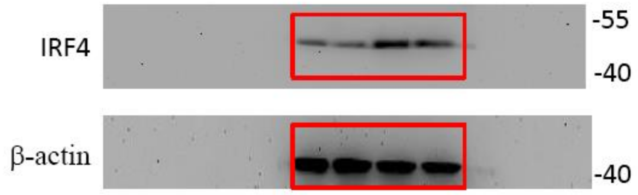

FigS6b

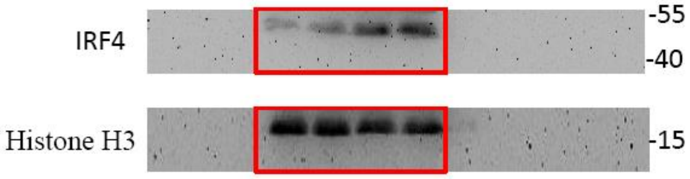

Uncropped scans of western blots displayed in the main figures

FigS7b

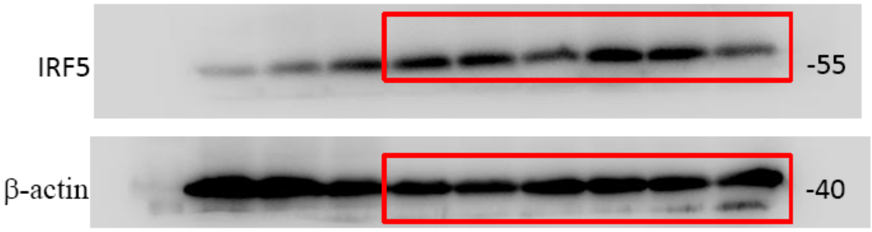

Uncropped scans of western blots displayed in the main figures

FigS8a

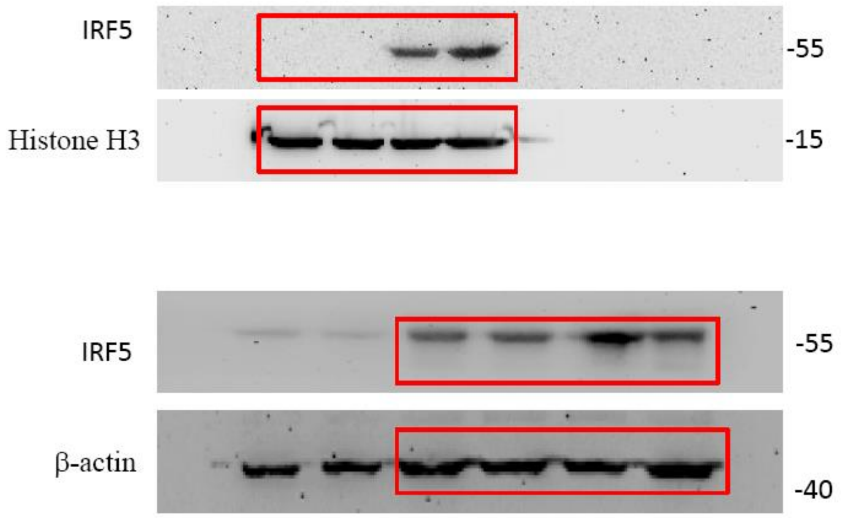

Uncropped scans of western blots displayed in the main figures

FigS8b

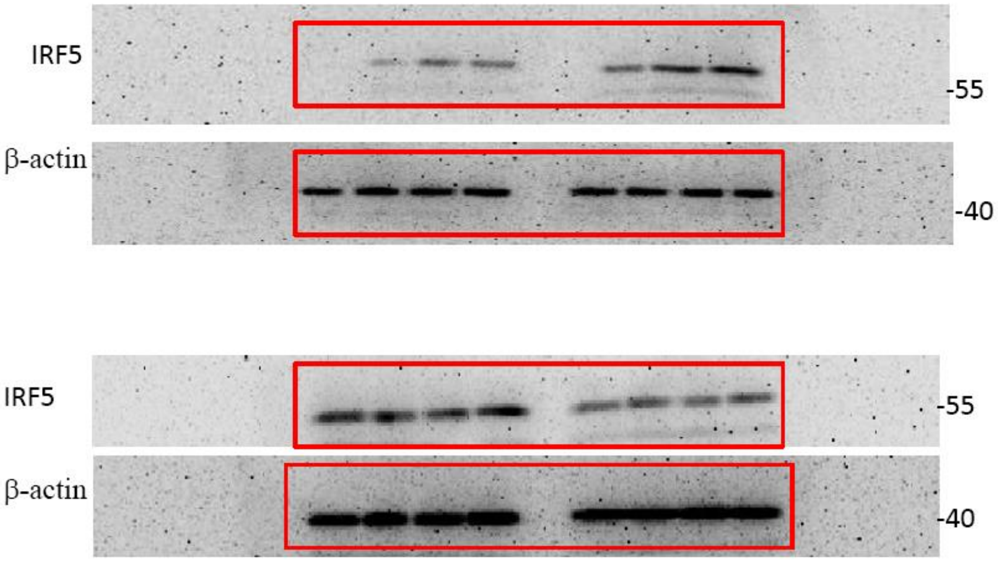

Uncropped scans of western blots displayed in the main figures

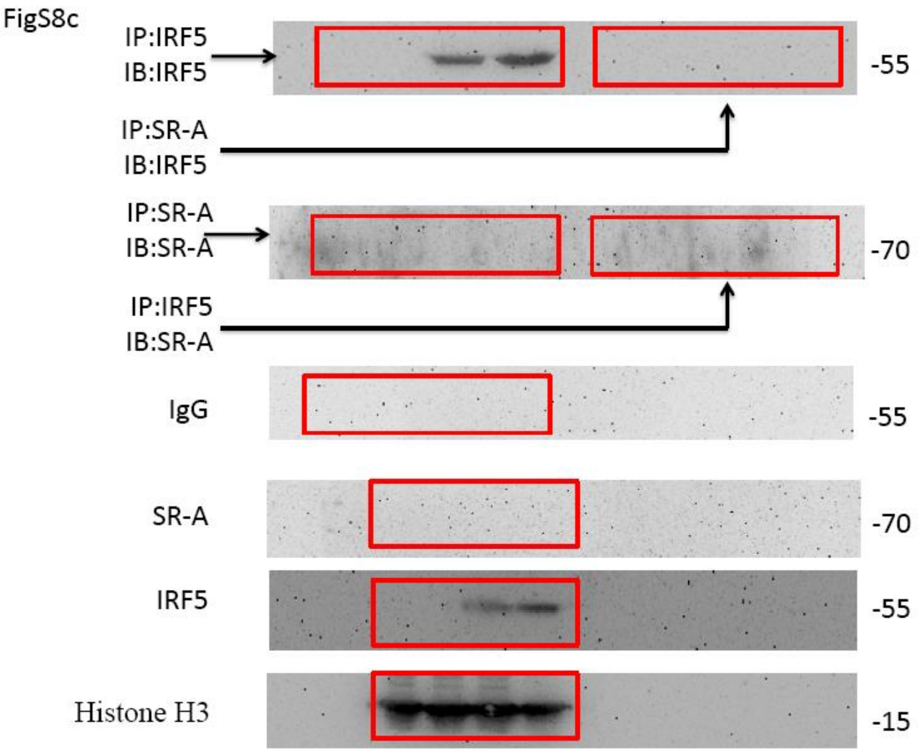

Uncropped scans of western blots displayed in the main figures

FigS8d

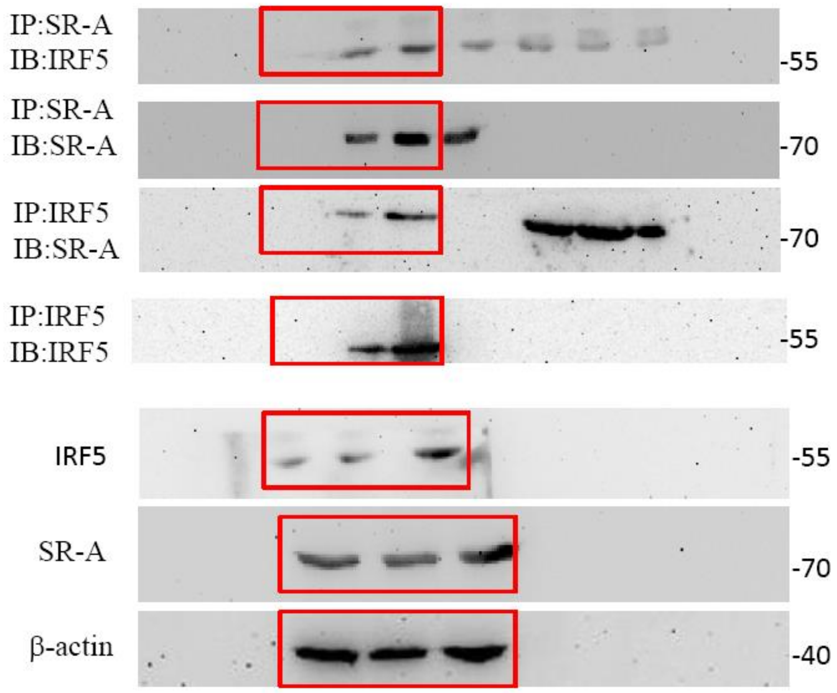

Uncropped scans of western blots displayed in the main figures

FigS8e

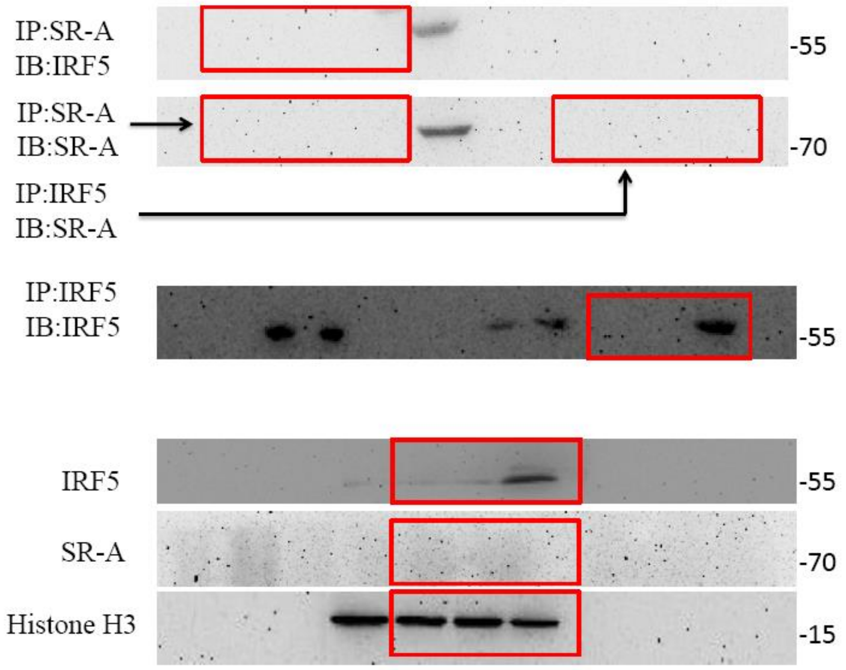

Uncropped scans of western blots displayed in the main figures

FigS8g

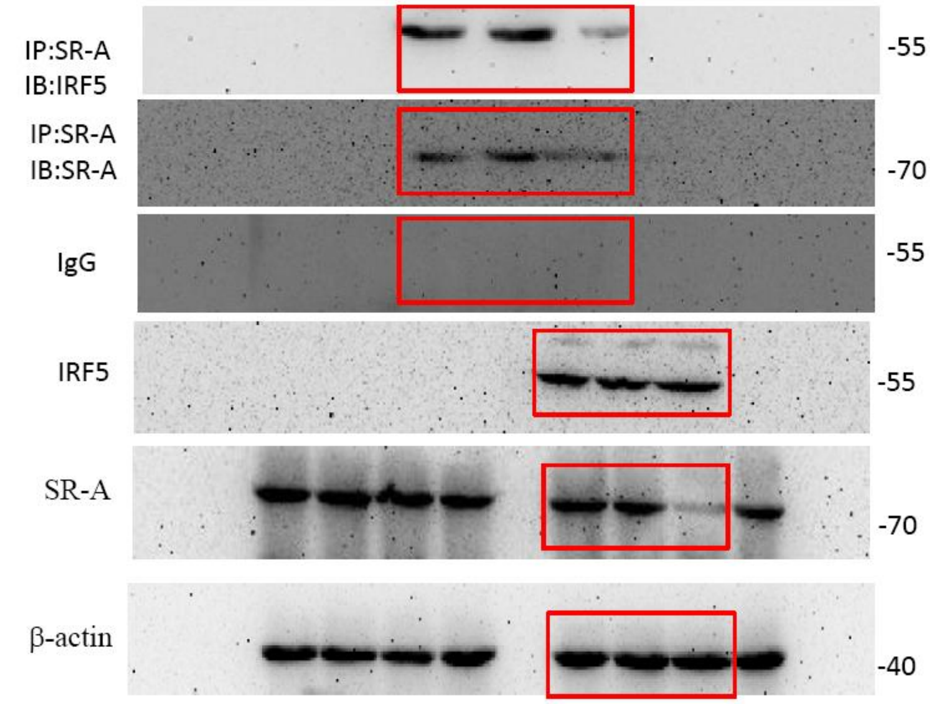

Uncropped scans of western blots displayed in the main figures

FigS8h

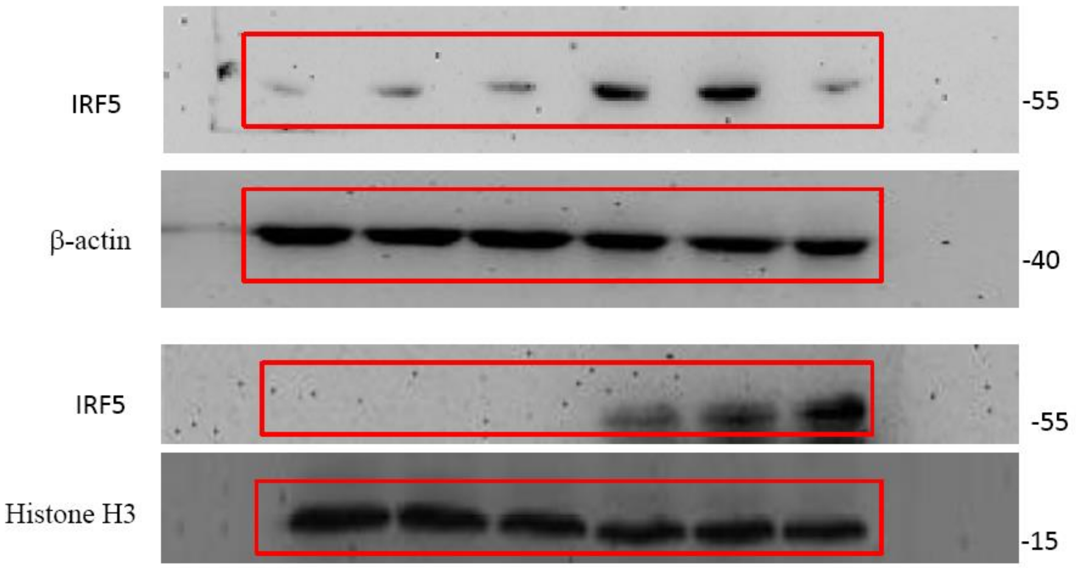

Uncropped scans of western blots displayed in the main figures

FigS8j

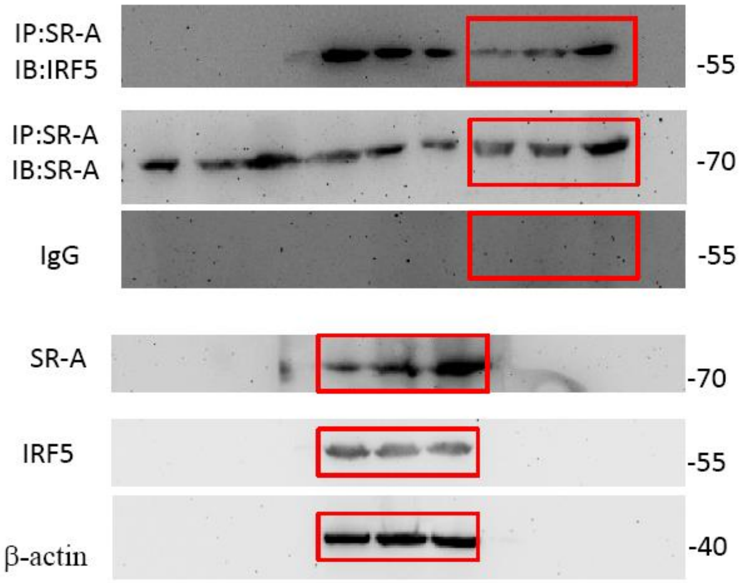

Uncropped scans of western blots displayed in the main figures

FigS8k

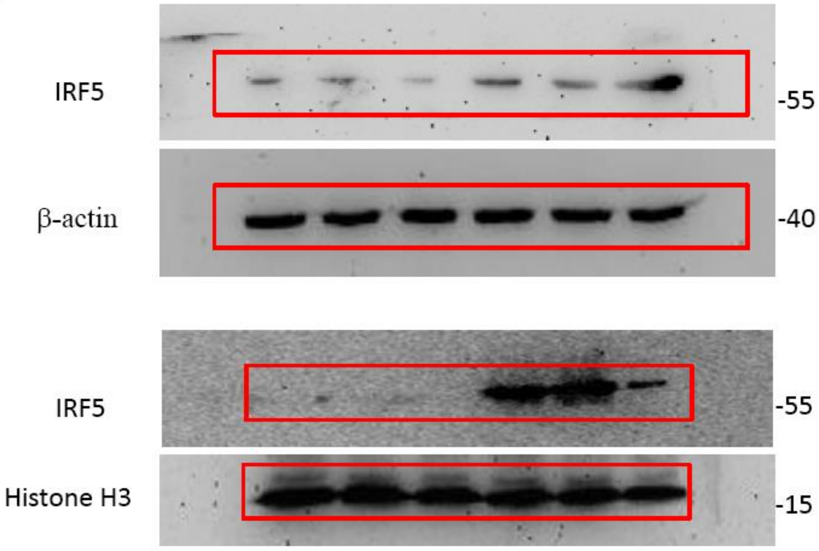

Uncropped scans of western blots displayed in the main figures

FigS10a

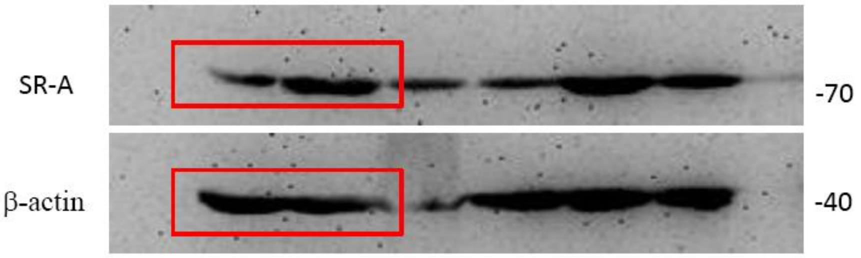

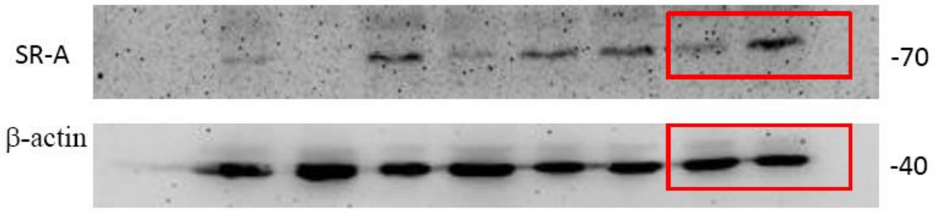

Uncropped scans of western blots displayed in the main figures

FigS10c

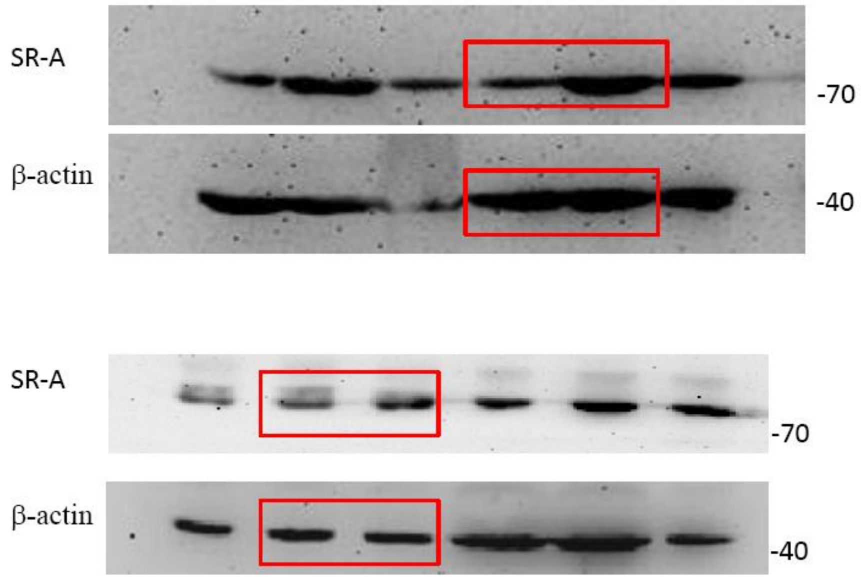

Uncropped scans of western blots displayed in the main figures

FigS10f

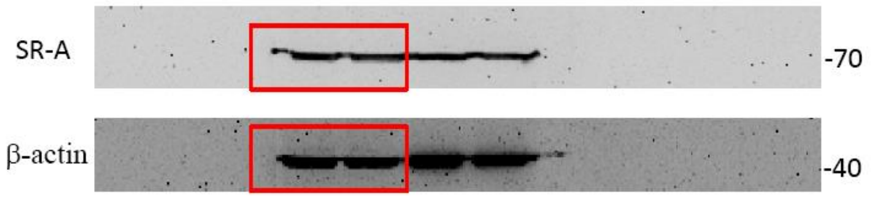

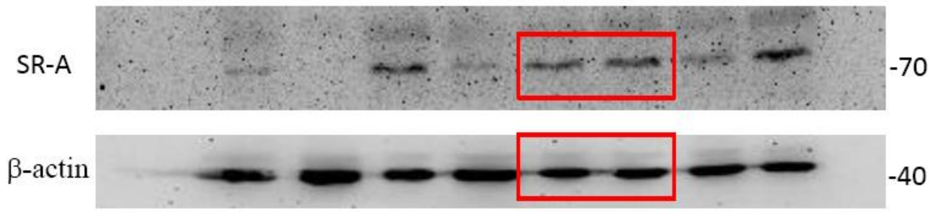

Uncropped scans of western blots displayed in the main figures

FigS10h

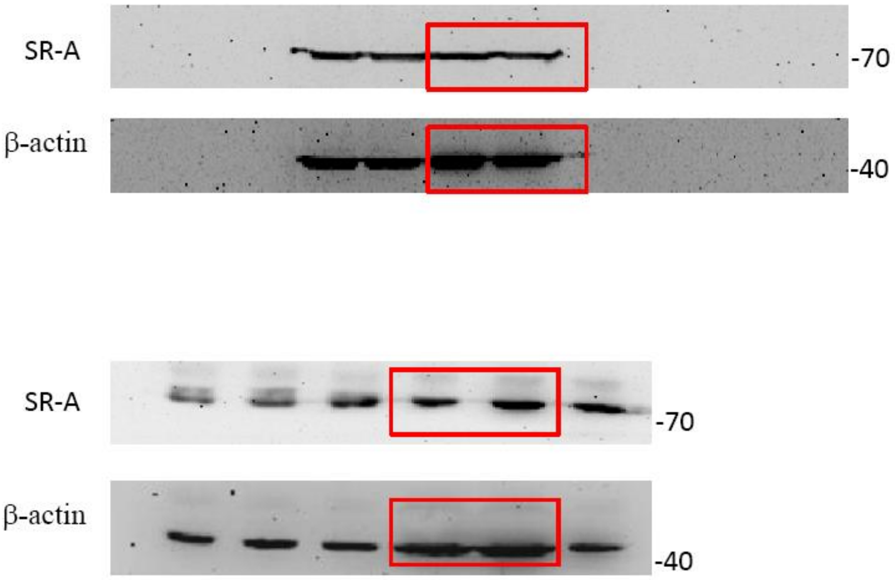

Uncropped scans of western blots displayed in the main figures

Supplementary Table 1. Sequence of Primers used

Gene name	forward	reverse
TNF	catcttctcaaaattcgagtgacaa	tgggagtagacaaggtacaaccc
IL-12p35:	gacagtggaggcaccaggcc	cagacatcgctgtcccggcg
IL-10	actttaagggttacttgggttgc	attttcacaggggagaaatcg
CXCL9	tctcggacttcactccaacaca	actccacactgctggaggaaga
CXCL10	ccgtcattttctgcctcatcc	ccctatggccctcattctca
CXCL11	gaacaggaaggtcacagccatagc	tcaactttgtcgcagccgttactc
CCL2	aagccagctctctcttcctcca	gcgttaactgcatctggctga
CCL17	agtgctgcctggattacttcaaag	ctggacagtcagaaacacgatgg
CCL22	taacatcatggctaccctgcg	tgtcttccacattggcacca
IL-4	acaggagaagggacgccat	gaagccctacagacgagctca
iNOS	gccaccaacaatggcaaca	cgtaccggatgagctgtgaatt
Arg-1	cagaagaatggaagagtcag	cagatatgcagggagtcacc
Ym-1	tcacaggtctggcaattcttctg	tttgtccttaggagggcttcctc
IL-6	gaggataccactcccaacagacc	aagtgcatcatcgttgttcataca
IRF5	aataccccaccaccttttga	aataccccaccaccttttga
GAPDH	ggtgaaggtcggtgtgaacg	accatgtagttgaggtcaatgaagg
